# Polysulfones Prepared by Radical Ring-Opening Polymerization of Cyclic Sulfolane Derivatives: Density Functional Theory Calculations, Synthesis, Structure, and Polymer Reactions

**DOI:** 10.3390/ma18050928

**Published:** 2025-02-20

**Authors:** Keisuke Yamanishi, Eriko Sato, Akikazu Matsumoto

**Affiliations:** 1 Department of Applied Chemistry and Bioengineering, Undergraduate School of Engineering, Osaka City University, 3-3-138, Sugimoto, Sumiyoshi-ku, Osaka 558-8585, Japan; 2 Department of Applied Chemistry and Bioengineering, Graduate School of Engineering, Osaka City University, 3-3-138, Sugimoto, Sumiyoshi-ku, Osaka 558-8585, Japan; sato-eriko@omu.ac.jp; 3 Department of Chemistry and Bioengineering, Graduate School of Engineering, Osaka Metropolitan University, 3-3-138, Sugimoto, Sumiyoshi-ku, Osaka 558-8585, Japan; 4 Department of Applied Chemistry, Graduate School of Engineering, Osaka Metropolitan University, 1-1, Gakuen-cho, Naka-ku, Sakai, Osaka 599-8531, Japan

**Keywords:** cyclic monomer, degradable polymer, polymer reaction, polysulfone, radical ring-opening polymerization, sulfolane, sulfolene

## Abstract

Recently, the radical ring-opening polymerization of cyclic monomers has become one of the most important topics because it can impart degradability to vinyl polymers by introducing functional groups and heteroatoms into the main chain through copolymerization with vinyl monomers. In this study, we investigated the possibility of the ring-opening polymerization of the cyclic sulfone compounds, including sulfolane and sulfolene derivatives. First, the reactions of 2,5-dimethyl-3-sulfolene (DMS) were predicted using density functional theory (DFT) calculations, and the reaction product was actually examined after heating in the presence of a radical initiator. Next, we synthesized and polymerized 2-vinylsulfolane (2VS), which has been reported to undergo radical ring-opening polymerization in the literature and tried to modify the resulting polymers containing the unsaturated groups in the main chain via post-polymerization reaction by ene–thiol additions. We also examined the decomposition behavior of the polymer of 2VS before and after hydrogenation. Furthermore, we discussed the reactivity of 3-exomethylenesulfolane (3EMS), which is expected to exhibit radical ring-opening polymerization similar to 2VS, as well as the structure and reactivity of the corresponding polymer.

## 1. Introduction

**Scheme 1:**
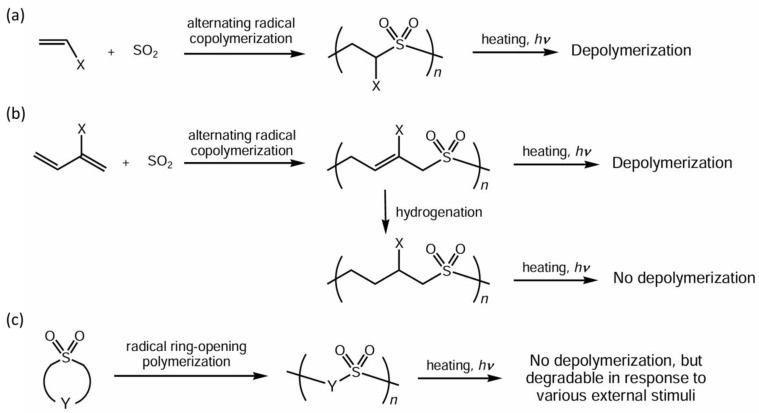
Synthesis of (**a**) poly(olefin sulfone)s and (**b**) poly(diene sulfone)s by the radical copolymerization of olefin and diene monomers with SO_2_ and degradation by heating and electron-beam radiation, and (**c**) polysulfones by the radical ring-opening polymerization of cyclic sulfones.

Polysulfones are polymers containing sulfonyl groups (−SO_2_−) in the repeating structure of the main chain, and they are classified into aromatic polysulfones and aliphatic polysulfones. Aromatic polysulfones have a main chain skeleton in which benzene rings are linearly bonded to sulfonyl groups or other functional groups (carbonyl groups, ether bonds, etc.) and are synthesized by the polycondensation of low-molecular-weight functional compounds [1]. For example, polysulfone, poly(phenylele sulfone), and poly(ether sulfone) are all amorphous and transparent engineering plastics with a high glass transition temperature (*T*_g_) around 200 °C. They also exhibit excellent properties such as hardness, heat resistance, and hydrolysis resistance [2]. On the other hand, aliphatic polysulfones are synthesized by the radical alternating copolymerization of olefin or diene monomers and sulfur dioxide and are called poly(olefin sulfone)s [3,4,5,6,7] and poly(diene sulfone)s [8,9,10,11], respectively (Scheme 1a,b). Poly(olefin sulfone)s are easily decomposed by heating or electron-beam irradiation, and radicals generated on the polymer main chain by irradiation cause a chain reaction that decomposes into low-molecular-weight products including the starting monomers [3,4,5,6,7]. Therefore, it can be used as an electron-beam resistance material. The photoinduced depolymerization of poly(olefin sulfone)s, the dismantlable adhesion materials, using photobase generators under UV irradiation was also applied [12,13,14]. Poly(olefin sulfone)s are useful as the customizable degradable polymers for various applications [15,16,17,18]. Poly(diene sulfone)s, like poly(olefin sulfone)s, are also easily decomposed by heating, but when the double bonds in the main chain are hydrogenated, the process of depolymerization is suppressed, and consequently, thermal stability is greatly improved (Scheme 1b) [8,9,10,11].

Radical ring-opening polymerization is a polymerization method that utilizes the ring-opening reaction of cyclic monomers, and the polymerization of vinylcyclopropane derivatives and other cyclic monomers containing exomethylene groups has been studied in detail [19,20,21,22,23]. Ring-opening polymerization allows for the introduction of functional groups into the main chain, and copolymerization with vinyl monomers can impart degradability to vinyl polymers that are poorly degradable. In recent years, therefore, many researchers have been conducting research on the radical ring-opening polymerization of cyclic monomers including ketene acetals [24,25,26,27,28,29,30,31,32,33] and thionolactones [34,35,36,37,38,39,40,41]. Sulfolane and sulfolene, which are cyclic sulfone compounds, are easy to synthesize and are stable compounds, so they are often used as solvents. If polysulfones are synthesized by the radical ring-opening polymerization of cyclic sulfones, polymerization would be possible without the need to use sulfur dioxide, a toxic gaseous monomer, in the polymerization process, unlike previous aliphatic polysulfone syntheses. It is also expected that this method will not only be able to synthesize a polymer with excellent thermal stability; it will also exhibit no depolymerizability (Scheme 1c). Polysulfones prepared by the radical ring-opening polymerization of cyclic sulfones may become one of the environmentally friendly polymers degradable in response to various external stimuli. When any unsaturated cyclic sulfones are polymerizable via a ring-opening polymerization mechanism, both sulfonyl groups and unsaturated groups are introduced into the main chain of the resulting polymers. It can be a new method for synthesizing polymers useful for polymer functionalization and recycling.

About 10 years ago, we began research on polysulfone synthesis using radical ring-opening polymerization [42]. At that time, reports on the synthesis of polysulfone by radical ring-opening polymerization were limited [43,44,45], and since then, only one report has been published [46]. In order to develop a solution to environmental problems such as microplastics and marine plastics [47] surrounding synthetic polymers, it is necessary to introduce functional groups and heteroatoms into the vinyl polymer main chain through radical ring-opening polymerization [19,20,21,22,23,48,49,50]. We considered that re-evaluating our previous experimental results will help in future polymer synthesis designs; therefore, we have decided to reorganize and discuss, again, the data that we have kept undisclosed.

**Figure 1:**
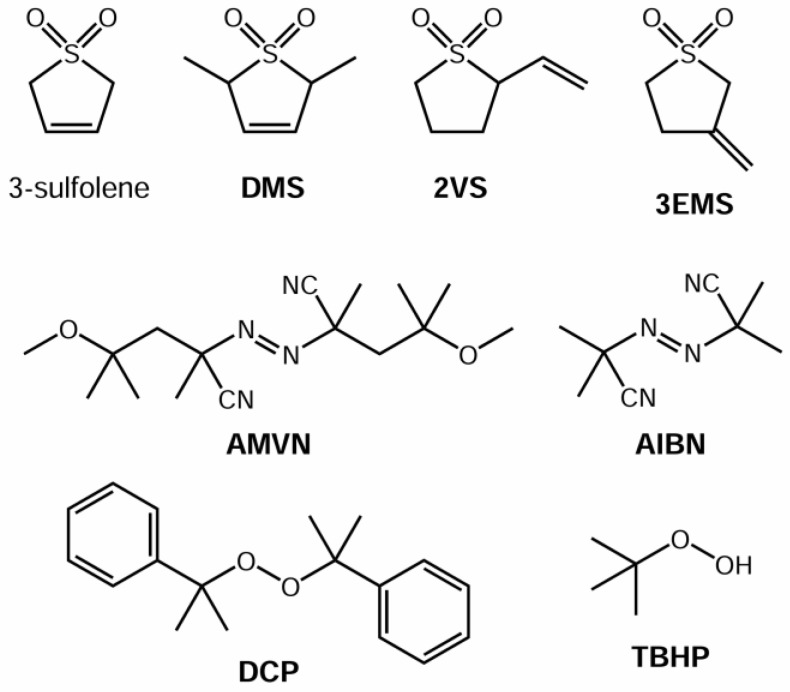
Structure of cyclic sulfone monomers, radical initiators, and the other reagents used in this study.

In this paper, the reaction prediction was first performed using density functional theory (DFT) regarding the possibility of ring-opening polymerization of 2,5-dimethyl-3-sulfolene (DMS) as the cyclic unsaturated sulfone compound (Figure 1). We attempted the radical polymerization of DMS in the presence of radical initiators at various temperature conditions. In addition, the reaction product was directly examined by NMR spectroscopy after heating. Next, we synthesized and polymerized 2-vinylsulfolane (2VS), which has already been reported to exhibit ring-opening polymerization reactivity [43,44,45,46], and characterized the resulting polymer containing the unsaturated groups in the main chain to investigate the functionalization and decomposition behavior of polymers. Furthermore, we also discussed the polymerization mechanism of 3-exomethylenesulfolane (3EMS), which is also expected to exhibit high ring-opening polymerization similar to 2VS, based on the results of the DFT calculations.

## 2. Experimental Section

### 2.1. Materials

Diisopropylamine (>98%), acetonitrile (>98%), toluene (>99%), 1,2-diethoxyethane (>97%), 1,2-dichloroethane (>99.5%), chloroform (>99.5%), dichloromethane (>99.5%), and methanol (1st grade) were purchased from Wako Pure Chemical Industries (Osaka, Japan) and used after distillation purification. Sulfur dioxide (Sumitomo Seika Co., Ltd., Osaka, Japan, >99.9 vol%) was liquefied and collected from a storage cylinder at −78 °C. 2,4-Hexadiene (HD, >95%, isomeric mixture), trimethylene sulfide (>98%), and 1-decanethiol (>95%) were purchased from Tokyo Chemical Industry Co., Ltd., Tokyo, Japan (TCI) and used as received. Allyl bromide (>97%), sodium periodate (>99.5%), *p*-toluenesulfonyl hydrazide (>90%), trifluoroacetic acid (TFA), 3-sulfolene (>98%), hydrochloric acid (1st grade), sodium bicarbonate (1st grade), and anhydrous sodium sulfate (1st grade) were purchased from Wako Pure Chemical Industries and used as received. *n*-Butyl lithium (*n*-hexane solution, 1.6 mol/L, manufactured by Kanto Kagaku, Co., Ltd., Tokyo, Japan) and *tert*-butyl hydroperoxide (TBHP, Tokyo Sigma-Aldrich Japan, Co., Ltd., Tokyo, Japan) were used as received. 2,2′-Azobis(isobutyronitrile) (AIBN, >98.0%, Wako Pure Chemical Industries), 2,2′-azobis(4-methoxy-2,4-dimethylvaleronitrile) (AMVN, >95.0%, Wako Pure Chemical Industries), and dicumyl peroxide (DCP, 98%, Sigma-Aldrich, Tokyo, Japan) were recrystallized with methanol before use. Nitrogen (>99.9 vol%) and Argon (>99.99 vol%) were purchased from Air Water, Inc., Osaka, Japan.

### 2.2. Synthesis

#### 2.2.1. Synthesis of 2.5-Dimethyl-3-Sulfolene (DMS)

Into a Pyrex ampoule (ca. 250 mL), 0.41 g (0.005 mol) of HD was added and removed dissolved oxygen under reduced pressure at −78 °C for 5 min. Then, sulfur dioxide (0.05 mol) was added by vacuum distillation, then the ampoule was sealed. After allowing the mixture to react at room temperature for 15 h, the mixture was cooled with dry ice, the ampoule was opened, and the mixture was poured into methanol. After filtering precipitate, the solvent in the filtrate was distilled off under reduced pressure, and the product was finally isolated using preparative GPC. The yield was 31.3%. The chemical structure of the product was confirmed by ^1^H and ^13^C NMR spectroscopy. The data are shown below.

DMS: solid; ^1^H NMR (300 MHz, CDCl_3_) δ5.94 (s, CH=CH, 2H), 3.80 (q, J = 6.9 Hz, CH_3_CH, 1H), 1.42 (d, CH_3_CH, J = 6.9 Hz, 3H);^13^C NMR (75 MHz, CDCl_3_) δ129.72 (CH=CH), 59.73 (*C*H), 13.65 (CH_3_).

#### 2.2.2. Synthesis of 2-Vinylsulfolane (2VS)

**Scheme 2:**
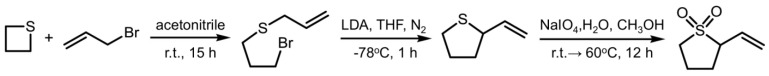
Reactions for the synthesis of 2VS. LDA: lithium diisopropylamide.

2VS was synthesized according to the reaction pathway in Scheme 2 [43,51].

##### Synthesis of Allyl-3-bromopropylsulfide [51]

In a 50 mL flask, 5.15 g (0.07 mol) of trimethylene sulfide, 8.39 g (0.07 mol) of allyl bromide, and 26 mL of acetonitrile were mixed, stirred at room temperature for 12 h, and then the solvent was distilled off under reduced pressure to obtain a product. Yield: 79.4%. The structure was confirmed by ^1^H and ^13^C NMR spectroscopy.

Allyl-3-bromopropylsulfide: liquid; ^1^H NMR (300 MHz, CDCl_3_) δ5.83–5.72 (m, CH=CH_2_, 1H), 5.15–5.08 (m, CH=CH_2_, 2H), 3.51 (t, J = 6.3 Hz, CH_2_Br, 2H), 3.16–3.12 (m, CH_2_CH=CH_2_, 2H), 2.61 (t, J = 6.9 Hz, CH_2_CH_2_S, 7 2H), 2.10 (tt, J = 6.9 Hz and 6.3 Hz, CH_2_CH_2_CH_2_, 2H); ^13^C NMR (75 MHz, CDCl_3_) δ134.32 (CH=CH_2_), 117.32 (CH=CH_2_), 34.84 (CH_2_), 32.29 (CH_2_), 32.15 (CH_2_), 28.88 (CH_2_).

##### Synthesis of 2-Vinylthiolane [43]

In a nitrogen atmosphere, 25 mL (0.004 mol) of *n*-butyllithium was added dropwise to 4.10 g (0.041 mol) of diisopropylamine, then tetrahydrofuran (THF) was added, and the mixture was stirred at −78 °C. Then, allyl-3-bromopropylsulfide 5.97 g (0.030 mol) was added dropwise over 30 min. After stirring for 1 h, ion-exchange water was added to stop the reaction, and after rising to room temperature, the mixture was washed with a 1 mol/L hydrochloric acid aqueous solution and a saturated sodium bicarbonate aqueous solution, and the organic layer was dried over anhydrous sodium sulfate. The solvent was distilled off under reduced pressure, and the resulting crude product was distilled under reduced pressure (50 °C, 2 mmHg) to obtain 2-vinylthiolane. Yield 79.9%.

2-Vinylthiolane: liquid; ^1^H NMR (300 MHz, CDCl_3_) δ5.86–5.74 (m, CH=CH_2_, 1H), 5.12 (d, J = 16.8 Hz, CH=CH_2_ (trans), 1H), 4.95 (d, J = 9.6 Hz, CH=CH_2_ (cis), 1H), 3.92 (dt, J = 9.6 and 5.7 Hz, SCH, 1H), 3.01–2.81 (m, CH_2_S, 2H), 2.22–1.60 (m, CH_2_CH_2_, 4H); ^13^C NMR (75 MHz, CDCl_3_) δ140.15 (CH=CH_2_), 114.58 (CH=CH_2_), 51.59 (SCH), 37.96 (*C*H_2_CH), 33.22 (CH_2_S), 31.00 (CH_2_*C*H_2_CH_2_).

##### Synthesis of 2VS [43]

At −10 °C, 40 mL of a methanol solution of 1.01 g (0.010 mol) of 2-vinylthiolane was added dropwise to 40 mL of an aqueous solution of 4.32 g (0.020 mol) of sodium periodate, and the mixture was stirred at room temperature for 10 h. After stirring at 60 °C for an additional 2 h, the precipitate was removed by filtration, extracted with dichloromethane, and dried over anhydrous sodium sulfate. The solvent was distilled off under reduced pressure, and the resulting crude product was fractionated by preparative GPC and further purified by column chromatography (developing solvent: ethyl acetate) to obtain 2VS. Yield 42.1%.

2VS: liquid; ^1^H NMR (300 MHz, CDCl_3_) δ5.86–5.73 (m, CH=CH_2_, 1H), 5.48–5.39 (m, CH=CH_2_, 2H), 3.62–3.56 (m, SO_2_CH, 1H), 3.19–2.98 (m, CH_2_SO_2_, 2H), 2.41–2.02 (m, CH_2_CH_2_, 4H); ^13^C NMR (75 MHz, CDCl_3_) δ128.62 (CH=CH_2_), 122.67 (CH=CH_2_), 65.26 (SO_2_CH), 50.80 (CH_2_SO_2_), 29.23 (CH_2_*C*H), 20.22 (CH_2_*C*H_2_CH_2_).

### 2.3. Polymerization

#### 2.3.1. Ring-Opening Polymerization of Cyclic Sulfones

In a Pyrex ampoule, the monomer, initiator, and solvent were placed ([monomer] = 1.0 mol/L, [initiator] = 0.01 mol/L), and the ampoule was sealed after the freeze–degassing–thaw cycle to remove dissolved oxygen. After the reaction was carried out at 60 °C for 18 h, it was cooled with dry ice, and the contents of the ampoule were poured into a large amount of methanol. The precipitate was filtered, washed, and then dried under vacuum at room temperature. It was reprecipitated and purified using trifluoroacetic acid and methanol as a solvent and a precipitant, respectively. The structure of the produced polymer was confirmed by ^1^H NMR, ^13^C NMR, and IR spectra.

P2VS: solid; ^1^H NMR (300 MHz, TFA-*d*_1_) δ6.16–5.96 (SO_2_CH_2_CH=CH, 1H), 5.84–5.64 (SO_2_CH_2_CH=CH, 1H), 4.19–3.81 (SO_2_CH_2_CH, 2H), 3.48–3.09 (SO_2_CH_2_CH_2_, 2H), 2.60–2.28 (CHCH_2_CH_2_, 2H), 2.24–1.92 (CH_2_CH_2_CH_2_, 2H); ^13^C NMR (75 MHz, TFA-*d*_1_) δ143.21, 141.13 (SO_2_CH_2_CH=CH), 117.85, 116.60 (SO_2_CH_2_CH=CH), 58.45, 53.70 (SO_2_CH_2_CH), 53.00 (SO_2_CH_2_CH_2_), 32.63, 27.92 (CHCH_2_CH_2_), 22.24 (CH_2_CH_2_CH_2_). IR (KBr) 3438, 2925, 1632, 1453, 1407, 1296, 1119, 975, 888, 734 cm^−1^.

#### 2.3.2. Radical Copolymerization of HD and Sulfur Dioxide

HD, TBHP, and toluene were placed in a Pyrex ampoule, and dissolved oxygen was removed by repeating the freeze–degassing–thaw cycle three times. Then, sulfur dioxide was added by vacuum distillation, and the tube was sealed. Here, the concentration ratio was [HD]/[SO_2_]/[TBHP] = 100/250/1 in a mol ratio. After polymerization was performed at −78 °C for 24 h, the mixture was cooled with dry ice and poured into methanol. The obtained copolymer, i.e., P(HD-*alt*-SO_2_), was filtered, washed, and then dried under vacuum at room temperature. It was reprecipitated and purified using chloroform and methanol.

### 2.4. Polymer Reaction of Polysulfones

#### 2.4.1. Thiol Addition

In a Pyrex ampoule, 25 mg of the polymer, 1-decanethiol or 1-butanethiol (5 eq. to the carbon–carbon double bond in the polymer), AIBN (0.5 eq.), and 2 mL of the solvent (TFA or 1,2-dichloroethane) were added. After repeated freezing–degassing–thawing cycles, the ampoule was sealed. The reaction was carried out at 60 °C for 18 h then cooled with dry ice, and the contents of the ampoule were poured into methanol. The precipitate was filtered, washed, and then dried under vacuum at room temperature. Reprecipitation was performed for purification. The thiol addition efficiency was determined from the integral ratio of ^1^H NMR spectrum.

#### 2.4.2. Hydrogenation

The polymer (0.05 g) and *p*-toluenesulfonyl hydrazide (0.93 g, 0.005 mol) were stirred in 1,2-diethoxyethane (5 mL) at 100 °C for 12 h under a nitrogen stream. After the reaction, the solution was poured into methanol. The precipitate was filtered, washed, and then dried under vacuum at room temperature. It was purified by reprecipitation using TFA and methanol. The hydrogenation efficiency was determined by ^1^H NMR spectroscopy.

### 2.5. Measurements

NMR measurements were performed at room temperature using a Bruker Avance 300 (Billerica, MA, USA). A JASCO FT/IR-430 spectrometer was used for IR measurement by the KBr method. Thermogravimetric analysis (TGA) was performed using Seiko Instruments TGA/DTA6200 (Tokyo, Japan) under a nitrogen stream (200 mL/min) at a heating rate of 10 °C/min. The maximum decomposition temperature (*T*_max_) was determined by the maximum value of the derivate curve. Seiko Instruments DSC6200 (Tokyo, Japan) was used for differential scanning calorimetry (DSC), and the measurements were performed at a heating rate and cooling rate of 10 °C/min. DFT calculations were performed using Spartan’08 (Wavefunction, Inc., Irvine, CA, USA). The calculation targets include radical substitution reactions on sulfur atoms, radical substitution reactions on carbon atoms adjacent to sulfonyl groups, additions to carbon–carbon double bonds, and ring-opening reactions due to intramolecular radical transfer. The energy difference between the production system and the reaction system (Δ*E*_n_) was calculated. Conformation optimization was performed using molecular mechanics method, and structural optimization and energy calculations were performed at the B3LYP/6-311G* level.

## 3. Results and Discussion

### 3.1. Ring-Opening Reactivity of Sulfolene Derivatives

**Scheme 3:**
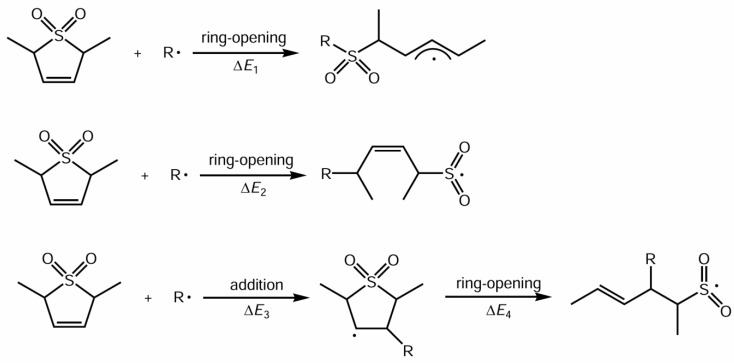
Possible reaction pathways including ring-opening and addition reactions of DMS with R.

In order to check ring-opening reactivity of sulfolene derivatives, DFT calculations were performed to estimate energy changes associated with some possible reaction pathways (Scheme 3). In this case, the radical addition to the double bond of DMS by a methyl radical and a methylsulfonyl radical, as well as the direct ring-opening reaction, were selected as the calculation targets. Among ring-opening reactions caused by the sulfonyl radical, the reaction in which a sulfur radical directly forms a new covalent bond with a sulfonyl group was excluded from the calculations because of its unreality. The results obtained are shown in Table 1. From these calculation results, only the addition of the methylsulfonyl radical to the double bond showed a positive value, and all other routes showed negative energy changes. This suggested the possibility of these reaction processes.

**Figure 2:**
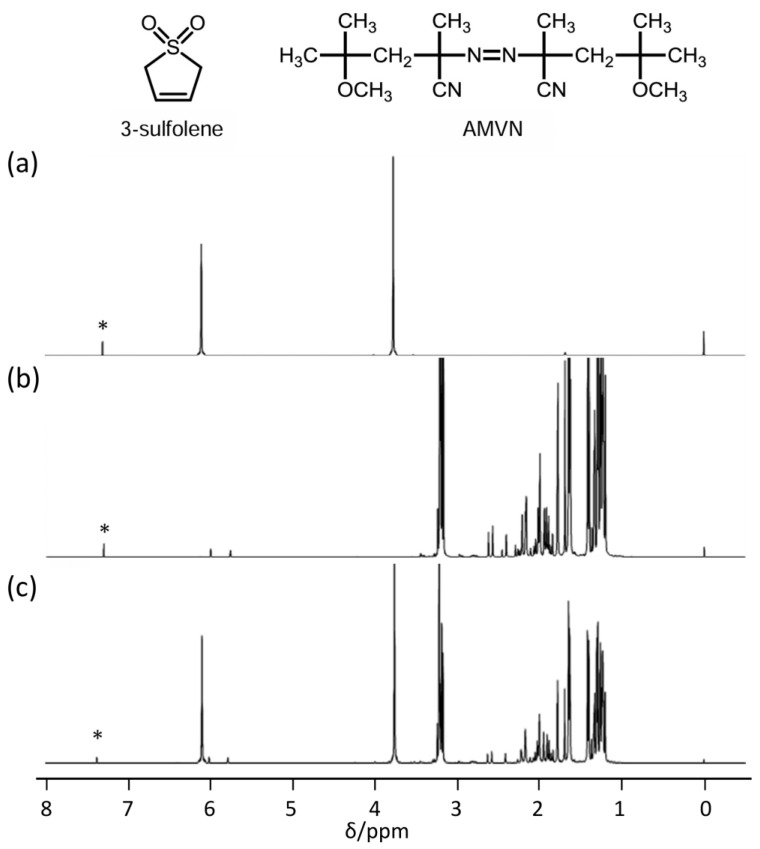
^1^H NMR spectra of the reaction systems of (**a**) 3-sulfolene, (**b**) AMVN, and (**c**) a mixture of 3-sulfolene and AMVN after heating at 50 °C for 2 h in CDCl_3_. Asterisk indicates a peak due to CHCl_3_.

Therefore, the polymerization of DMS was performed in toluene for 18 h at 30, 60, and 110 °C using AMVN, AIBN, and DCP as the radical initiators, respectively, under the conditions of [monomer] = 1.0 mol/L and [initiator] = 0.01 mol/L. However, contrary to expectations, no methanol-insoluble product could be obtained from any of the reaction systems. Furthermore, in order to directly confirm whether addition to the double bond was occurring or not, the solution after the reaction was directly analyzed under the conditions where the amount of an initiator was increased. Here, commercially available 3-sulfolene, which is thought to have reactivity similar to DMS, was used as a monomer. After reacting for 2 h at 50 °C under an argon atmosphere in CDCl_3_ at a concentration of [3-sulfolene] = [AMVN] = 0.5 mol/L, the product was identified by ^1^H NMR measurement of the solution (Figure 2). As a result, the ^1^H NMR spectrum of the reaction solution after heating was simply the sum of the spectra after heating each of 3-sulfolene and AMVN individually. Thus, radical addition to the sulfolene or a ring-opening reaction could not be confirmed.

As shown in Table 1, the Δ*E*_3_ for the addition of sulfonyl radicals is a positive value (+4.5 kcal/mol), while the values for the other reactions are negative. It means that the reactivity of the internal olefin-type double bond contained in the sulfolene compounds is low. This disadvantageous addition of an alkylsulfonyl radical to the internal olefin of an unsaturated cyclic sulfone is a main reason for no polymerization result. Here, if the addition to the double bond proceeds rapidly, ring-opening polymerization may be possible. The calculated values of Δ*E*_4_ for the ring-opening reaction are sufficient to proceed with the sequential reactions.

**Scheme 4:**
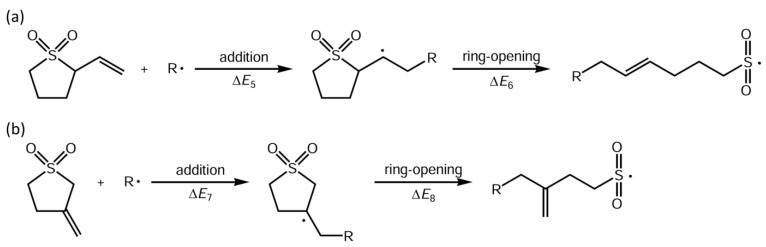
Addition and ring-opening reactions of (**a**) 2VS and (**b**) 3EMS with R·.

The above results revealed that in order to design a cyclic sulfone capable of ring-opening polymerization, it is necessary to devise the chemical structure of a monomer to increase the additional reactivity of the double bond. Therefore, we selected 2VS and 3EMS as monomer candidates, which are expected to exhibit high reactivity. Scheme 4 shows the radical addition and ring-opening reactions envisioned for 2VS and 3EMS, which each have a vinyl group and an exomethylene group. Additionally, Table 1 shows the results of DFT calculations for these reactions. Negative energy changes were observed for all reaction processes, including not only the addition of the methyl radical but also the addition of the sulfonyl radical. The values of Δ*E*_5_ and Δ*E*_7_ for the addition of the sulfonyl radical to the double bond of 2VS and 3EMS, which are assumed to be the rate-determining step, are also negative (−3.6 kcal/mol and −2.4 kcal/mol, respectively). It was suggested that either route of 2VS and 3EMS is capable of proceeding with polymerization.

### 3.2. Polymerization of 2VS

**Figure 3:**
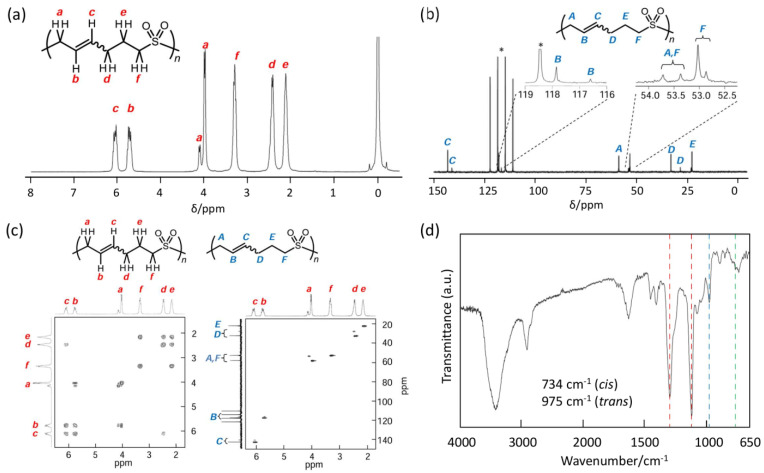
(**a**) ^1^H NMR, (**b**) ^13^C NMR, (**c**) ^1^H-^1^H COSY and HSQC, and (**d**) IR spectra of P2VS. The NMR spectra were observed in TFA-*d*_1_ at room temperature. Asterisk indicates peaks due to the solvent.

Regarding the polymerization of 2VS, it has already been reported that ring-opening polymerization is possible [43,44,45,46], but the post-polymerization reaction of the resulting polymers, including modification and functionalization, has not been investigated in detail. In this study, therefore, we first analyzed the structure of the polymer produced by the polymerization of 2VS. When polymerization was carried out in toluene under the conditions of [2VS] = 1.0 mol/L and [AIBN] = 0.01 mol/L at 60 °C, a white solid began to precipitate immediately after the reaction started, and the polymerization mixture lost fluidity after 30 min. After reacting for 18 h, the polymerization mixture was poured into methanol, and the resulting precipitate was filtered, washed with methanol, and then dried under vacuum at room temperature. Since the obtained polymer (P2VS) was soluble only in TFA, it was purified by reprecipitation using TFA and methanol. The isolated yield was 85%. The chemical structure of P2VS was identified from the ^1^H NMR, ^13^C NMR, 2D NMR (H-H COSY and HSQC) spectra, and IR spectrum (Figure 3).

**Scheme 5:**
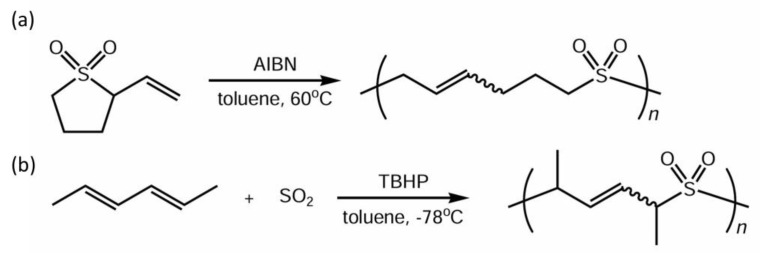
Synthesis of polysulfones by radical polymerization: (**a**) radical ring-opening polymerization of 2VS; (**b**) radical alternating copolymerization of HD with SO_2_.

The formation of polysulfone was confirmed from the S=O stretching vibrations observed at 1131 cm^−1^ and 1310 cm^−1^ in the IR spectrum. In addition, the peaks b and c observed in the ^1^H NMR spectrum, the peak C observed in the ^13^C NMR spectrum, and the absorption of the C-H out-of-plane displacement vibration of the alkene at 734 cm^−1^ and 975 cm^−1^ in the IR spectrum are all due to the presence of a carbon–carbon double bond in the repeating structure of the polymer, as shown in Scheme 5a. Thus, we confirmed that 2VS produces polymers not by vinyl polymerization (continuous double bond cleavage) but by radical ring-opening polymerization, which involves repeated addition and ring-opening. Furthermore, two types of peaks are observed for the absorption a in the ^1^H NMR spectrum and absorptions A-D in the ^13^C NMR spectrum indicate that the double bonds contained in the main chain of P2VS has both *cis* and *trans* isomeric structures. Absorptions derived from the cis and trans forms were also observed for C-H out-of-plane displacement vibrations in the IR spectrum. More recently, a reaction route for the synthesis of 3EMS containing an exomethylene group was newly proposed and preliminary experimental results for the monomer synthesis and its radical ring-opening polymerization were reported [52]. The results for the synthesis and polymerization of 3EMS will be separately reported in the future.

### 3.3. Polymer Reaction of P2VS

**Figure 4:**
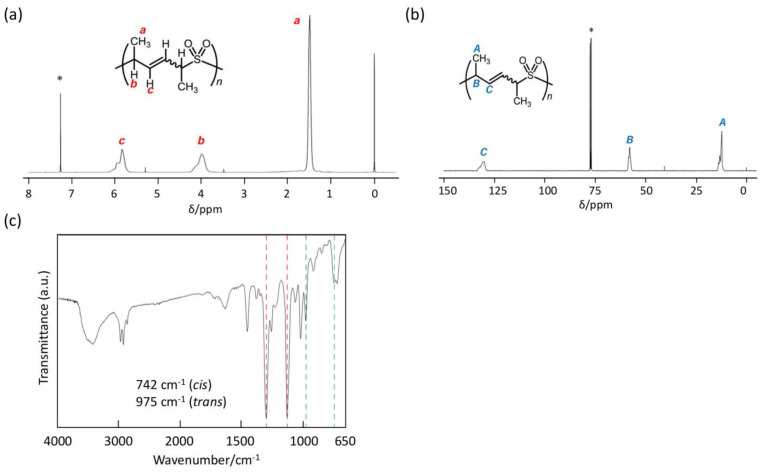
(**a**) ^1^H NMR, (**b**) ^13^C NMR, and (**c**) IR spectra of P(HD-*alt*-SO_2_). The NMR spectra were observed in CDCl_3_ at room temperature. Asterisk indicates peaks due to the solvents.

**Scheme 6:**
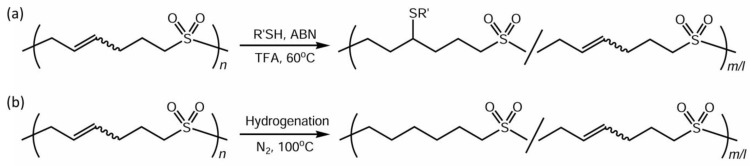
Functionalization of polysulfones produced by radical ring-opening polymerization and the post-polymerization reactions: (**a**) ene–thio addition; (**b**) hydrogenation with *p*-toluenesulfonyl hydrazide.

By performing addition reactions on the double bonds contained in the main chain of P2VS, various changes in physical properties are expected. In particular, thiol addition is one of the representative reactions of click chemistry [53,54,55] and is effective in improving solubility and the refractive index. In this study, we performed thiol addition as shown in Scheme 6a on P2VS, and the reactivity was compared with P(HD-*alt*-SO_2_) [8,9,10,11] (Scheme 5b). P(HD-*alt*-SO_2_) was synthesized according to the conditions shown in the experimental section and isolated with a yield of 93.7%. ^1^H NMR, ^13^C NMR, and IR spectra of P(HD-*alt*-SO_2_) are shown in Figure 4.

**Figure 5:**
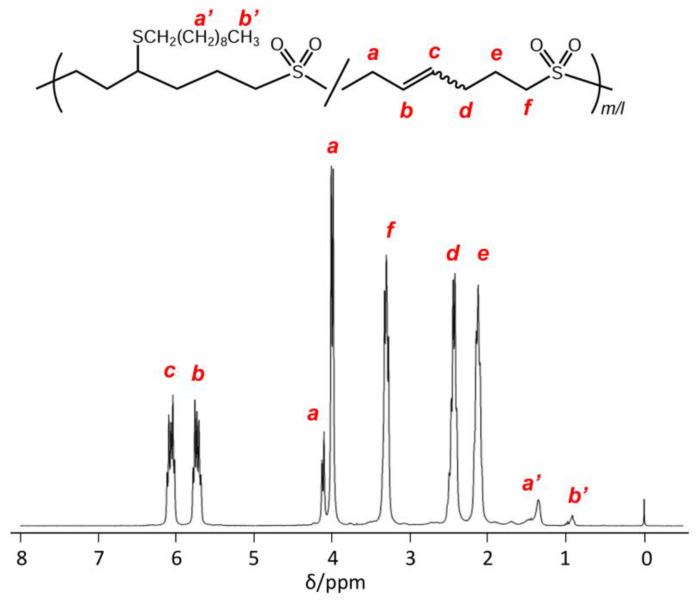
^1^H NMR spectrum of P2VS after reaction with 1-decanethiol. The NMR spectrum was observed in TFA-*d*_1_ at room temperature.

Table 2 shows the results of the thiol addition to these polymers containing unsaturated bonds in their main chains. After the thiol addition to P(HD-*alt*-SO_2_), no polymer could be recovered when the reaction solution was poured into methanol. It is thought that poly(diene sulfone) immediately depolymerized when radicals were generated on the main chain by the addition of the thiyl radical. On the other hand, P2VS was able to recover a polymer insoluble in methanol, and it was confirmed that some thiol molecules were added based on the ^1^H NMR spectrum (Figure 5) after reprecipitation and purification with TFA and methanol. However, the conversion was as low as 2%, which may be due to the internal olefin structure.

**Figure 6:**
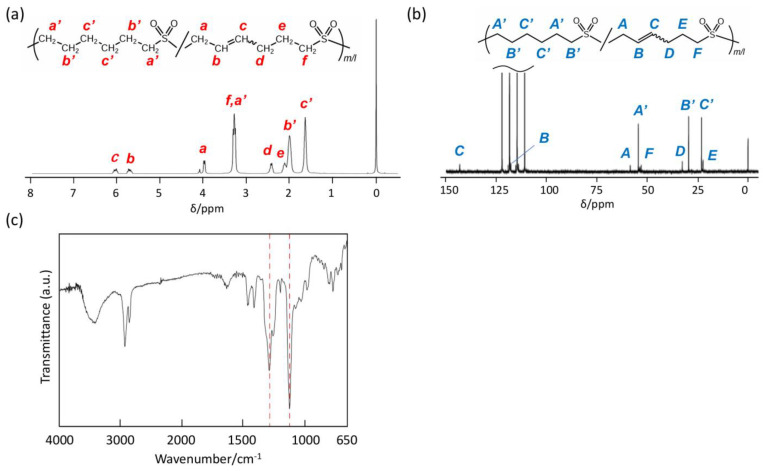
(**a**) ^1^H NMR, (**b**) ^13^C NMR, and (**c**) IR spectra of P2VS after hydrogenation with *p*-toluenesulfonyl hydrazide. The NMR spectra were observed in CDCl_3_ at room temperature.

**Figure 7:**
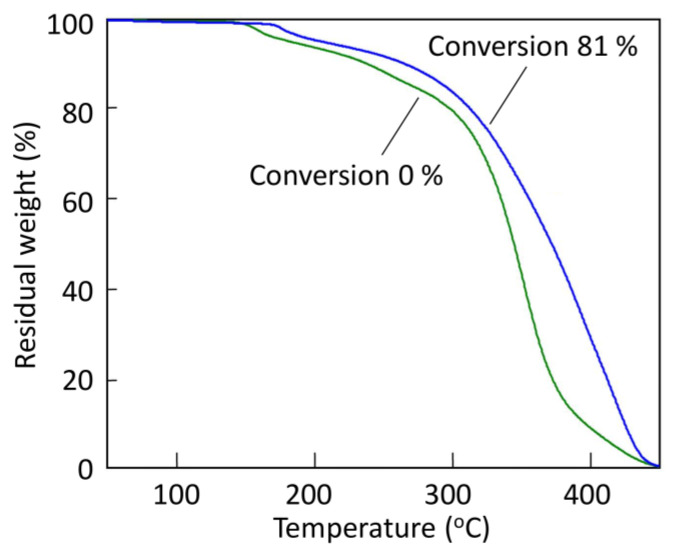
TGA curves for P2VS before and after hydrogenation with *p*-toluenesulfonyl hydrazide. Measurement conditions: in a nitrogen stream at a heating rate of 10 °C/min.

Poly(diene sulfone) has low thermal stability due to its depolymerization property, but it is known that the thermal stability can be improved by hydrogenating the double bonds contained in the main chain [8,9,10,11]. Since depolymerization does not occur for P2VS, it exhibits relatively high thermal stability. Also, like poly(diene sulfone)s, it has a double bond in its main chain. Therefore, hydrogenation was expected to further improve the thermal stability of P2VS [46], and we investigated changes in the repeating structure and the thermal properties of each polymer before and after the hydrogenation of P2VS using *p*-toluenesulfonyl hydrazide (Scheme 6b). Figure 6 shows the ^1^H NMR, ^13^C NMR, and IR spectra of P2VS after hydrogenation. The degree of progress of hydrogenation was confirmed from the ^1^H NMR spectrum after hydrogenation and was 81% and 98% for P2VS and P(HD-*alt*-SO_2_), respectively. TGA and DSC measurements were performed to examine the thermal properties of the polymers. Figure 7 shows the TGA curves before and after the hydrogenation reaction to P2VS. Table 3 shows the 5% weight loss temperature (*T*_d5_), the maximum decomposition temperature (*T*_max_), and the *T*_g_ of the polymer before and after the reaction of P2VS and P(HD-*alt*-SO_2_). P2VS showed high thermal stability even before hydrogenation, but the thermal stability was further improved by hydrogenation. For P2VS, hydrogenation can also be expected to improve performance in terms of oxidation stability and weather resistance. It is expected that the transformation and functionalization of P2EMS by polymer reactions will provide polymer material exhibiting properties different from those of P2VS, because P2EMS includes a different type of unsaturated carbon-to-carbon double bond in the main chain, as shown in Scheme 4b. This will be reported in the future.

## 4. Conclusions

We investigated the radical ring-opening polymerization of cyclic sulfone compounds. It was experimentally confirmed that sulfolene, as the unsaturated cyclic sulfone containing an internal olefin structure, does not exhibit ring-opening polymerization. Comparison of energy changes for each reaction pathway of the addition to double bonds and ring-opening reactions estimated by DFT calculations shows that ring-opening polymerization can be induced by introducing a vinyl group or an exomethylene group into a cyclic sulfone compound. Actually, we obtained a polymer in high yield by the radical ring-opening polymerization of 2VS. When we performed thiol addition and hydrogenation to the double bonds contained in the main chain of P2VS, we found that the hydrogenation proceeded with a conversion of 80% and further improved the thermal stability of the polymer, while the conversion for thiol addition was low. On the other hand, P(HD-*alt*-SO_2_), which has double bonds and sulfonyl groups in the main chain, similar to P2VS, had high depolymerization reactivity, and the polymer degraded upon addition of a thiol radical. Although the synthesis of polysulfone by the radical ring-opening polymerization of cyclic sulfone compounds have been extremely limited, we succeeded in the demonstration of the possibility of the radical ring-opening polymerization of 2VS and 2EMS via an actual experimental approach and the estimation of reactivity via the DFT calculations. This study offers a sustainable alternative to the traditional polysulfone synthesis because it can avoid the use of toxic sulfur dioxide and contribute to the development of recyclable, environmentally friendly polymers in the next generation. In order to solve environmental problems, including the proliferation of microplastics and marine plastics, we believe that the results obtained in this study may be of some help in the future.

## Figures and Tables

**Table 1:** Results for the DFT calculations for the reactions of DMS, 2VS, and 3EMS with alkyl and sulfonyl radicals ^a^.

Cyclic Monomer	Radical	Addition (kcal/mol)	Ring Opening (kcal/mol)
DMS	·CH_3_	-	Δ*E*_1_ = −26.5
	·SO_2_CH_3_	-	Not calculated
	·CH_3_	-	Δ*E*_2_ = −36.9
	·SO_2_CH_3_	-	Δ*E*_2_ = −8.6
	·CH_3_	Δ*E*_3_ = −26.4	Δ*E*_4_ = −10.0
	·SO_2_CH_3_	Δ*E*_3_ = +4.5	Δ*E*_4_ = −9.6
2VS	·CH_3_	Δ*E*_5_ = −30.1	Δ*E*_6_ = −8.6
	·SO_2_CH_3_	Δ*E*_5_ = −3.6	Δ*E*_6_ = −9.2
3EMS	·CH_3_	Δ*E*_7_ = −29.9	Δ*E*_8_ = −7.0
	·SO_2_CH_3_	Δ*E*_7_ = −2.4	Δ*E*_8_ = −5.7

^a^ See also Scheme 3 and Scheme 4 for each reaction.

**Table 2:** Results for the polymer modification of polysulfones using ene–thiol reactions at 60 °C for 18 h.

Polymer	Thiol	Solvent	Yield (%)	Conv. ^a^ (%)
P2VS	1-Decanethiol	TFA	51	2.1
P(HD-*alt*-SO_2_)	1-Butanethiol	1,2-Dichloroethane	0	Not determined

^a^ Determined by ^1^H NMR spectroscopy.

**Table 3:** Thermal properties of polysulfones before and after hydrogenation.

Polymer	Time (h)	Conv. ^a^ (%)	*T*_d5_ (°C)	*T*_max_ (°C)	*T*_g_ ^b^ (°C)
P2VS	0	0	187	353	^c^
	12	80.8	221	385	^c^
P(HD-*alt*-SO_2_)	0	0	135	235	^c^
	12	98.2	278	346	90.4

^a^ Determined by ^1^H NMR spectroscopy. ^b^ Determined by DSC. ^c^ Not observed in a temperature range 50 °C below the onset temperature of decomposition.

## Data Availability

The original data are based on the graduation thesis of Mr. Keisuke Yamanishi, and it is a private and non-public documents.
